# Nasal Cytology Changes in Head and Neck Cancer Treatment: A Systemic Review

**DOI:** 10.3390/diagnostics13152480

**Published:** 2023-07-26

**Authors:** Giuseppe Riva, Anastasia Urbanelli, Marta Trossarello, Federica Piazza, Giancarlo Pecorari

**Affiliations:** Division of Otorhinolaryngology, Department of Surgical Sciences, University of Turin, 10126 Turin, Italy; anastasia.urbanelli@unito.it (A.U.); marta.trossarello@unito.it (M.T.); f.piazza@unito.it (F.P.); giancarlo.pecorari@unito.it (G.P.)

**Keywords:** nasal cytology, head and neck cancer, head and neck surgery, radiotherapy, laryngectomy, rhinitis, radiation, chemotherapy

## Abstract

Nasal cytology is a non-invasive, low-cost exam that can help physicians in the diagnosis of allergic and nonallergic rhinitis, discriminating between different nasal disorders. The aim of this review is to summarize and analyze the current knowledge about nasal cytological examination in head and neck cancer, with a specific focus on the effects of different treatments. Indeed, nasal cytology is important to choose the best treatment for nasal complaints in each patient. A review of the English literature (PubMed, Scopus, Cochrane) was performed (5404 records screened). The inclusion criteria were clinical trials, cohort studies, case–control studies, case series, and case reports regarding nasal cytology in head and neck cancer treatment. Exclusion criteria were as follows: non-human studies, non-English literature, non-cytological evaluations. Two independent reviewers, working separately, extracted the data from all the eligible studies, which were subsequently cross-checked. Five studies were included in qualitative synthesis: three assessed mucosal disorders after radiation therapy and two after total laryngectomy. Radiotherapy can determine mucous or squamous cell metaplasia and neutrophil inflammation. Laryngectomees show hyperplasia of the basal zone cells and mucous cell metaplasia, and they do not develop inflammatory changes. The main limitation of this review is the low number and heterogeneity of studies present in the literature. In conclusion, nasal cytology is useful and allows for identifying mucosal disorders of the nasal cavities after surgery and/or radiotherapy for head and neck cancer. This can help physicians to better treat the nasal complaints of such patients.

## 1. Introduction

Head and neck cancer (HNC) is a heterogeneous group of malignancies and represents the sixth most common tumor worldwide, with an incidence in Europe of approximately 21.8 per 100,000 and a mortality rate of approximately 15.6 per 100,000 [1,2]. The major risk factors for head and neck squamous cell carcinoma (SCC) are smoking and alcohol consumption, and Human papillomavirus (HPV) has a key role in the etiology of oropharyngeal SCCs [3]. Despite innovations, HNC diagnosis is often late because of unspecific symptoms and patient delays in referring them to the doctor [4]. Currently, surgery is the main treatment for most HNCs. Chemotherapy (CT) and radiotherapy (RT) have a crucial role as exclusive or adjuvant treatments, based on tumor stage and the primary site involved [5]. Early-stage HNC may often be treated with surgery or exclusive RT, while a combination of surgery, RT, and CT is essential for locally advanced tumors. Indeed, patients affected by locally advanced tumors who undergo surgery are candidates for adjuvant RT or chemoradiotherapy (CRT). CT and immunotherapy are recommended for metastatic or recurrent HNC when surgery has been excluded [5]. At last, electrochemotherapy represents a palliative option for recurrent oral and oropharyngeal cancer [6,7]. Globally, the 5-year overall survival for all HNCs is about 50–60% [8].

The current gold standard for the diagnosis of a potentially malignant disorder or HNC is biopsy. In recent years, oral brush biopsy using liquid-based cytology emerged as a diagnostic tool for oral disorders [9]. Modified criteria adopted from the 2014 Bethesda System for Reporting Cervical Cytology were developed for clinical application in the oral cavity with an accuracy of 75%, with similar percentages for sensitivity and specificity [10].

Nasal cytology emerged in the past few decades as a new non-invasive, low-cost exam that can help physicians in the diagnosis of allergic and nonallergic rhinitis, discriminating between different nasal pathologies [11,12]. Healthy nasal mucosa is a pseudostratified epithelium lying on a basement membrane and it is composed of only four cytotypes: ciliated, goblet, striated, and basal cells. Nasal samples are generally obtained by scraping the medial portion of the inferior turbinates using an appropriate curette. Then, the sample is laid on a microscope slide, fixed for 4 s in 95° alcohol, and stained by the May–Grunwald–Giemsa method. Observation is performed by an optic microscope [13]. Nasal cytology allows for identifying and describing the epithelial and inflammatory cells and the infecting agents, such as bacteria and fungal hyphae/spores. Some specific cytological patterns are useful to discriminate between various diseases. Therefore, allergic, non-allergic, and infectious rhinitis and overlapping forms can be easily identified [11,14].

According to the predominant inflammatory cell type (lymphocytes, neutrophils, eosinophils, and/or mast cells), various pathological entities can be distinguished. In particular, non-allergic rhinitis is classified as eosinophilic (NARES), mast cellular (NARMA), mixed eosinophilic–mast cellular (NARESMA), or neutrophilic (NARNE) [13]. Moreover, nasal cytology performed with an optical microscope can play an important role in detecting biofilm, which is present not only in infectious rhinitis but also in inflammatory and/or immune-mediated diseases. Therefore, nasal cytology is particularly useful when signs and symptoms are not sufficient to distinguish the different rhinitis phenotypes [13].

The aim of this review is to summarize and analyze our current knowledge about the role of nasal cytological examination in HNC, with a specific focus on the effects of different treatments. The main objective was to investigate how nasal cytology may help clinicians in diagnosing and treating HNC patients.

## 2. Materials and Methods

A review of the English literature was performed through several databases (PubMed, Scopus, Cochrane, accessed on 31 December 2022) in order to identify articles published before 31 December 2022, according to Preferred Reporting Items for Systematic Reviews and Meta-Analyses (PRISMA) standards (Supplementary Materials). The primary search was performed using the terms “nasal cytology” and “cancer OR tumor OR radiotherapy”. Search strategies were adapted for each database: “(nasal OR nose) AND cytolog* AND (cancer OR tumor OR radiotherapy)” for PubMed; “(nasal cytology) AND (cancer OR tumor OR radiotherapy)” for Scopus and Cochrane.

The inclusion criteria were clinical trials, cohort studies, case–control studies, case series, and case reports regarding nasal cytology in head and neck cancer treatment. Exclusion criteria were as follows: non-human studies, non-English literature, non-cytological evaluations.

The abstracts of all suitable articles were examined using the inclusion criteria for applicability. The references of the selected publications were reviewed in order to identify further reports that were not found by database searching. Two independent reviewers (A.U., M.T.), working separately, extracted the data from all the eligible studies, which were subsequently cross-checked. All retrieved full-text articles were included in the review by a consensus of all the authors. Data about epithelial and inflammatory changes were extracted from the included studies. Results were reported as percentages.

**Figure 1:**
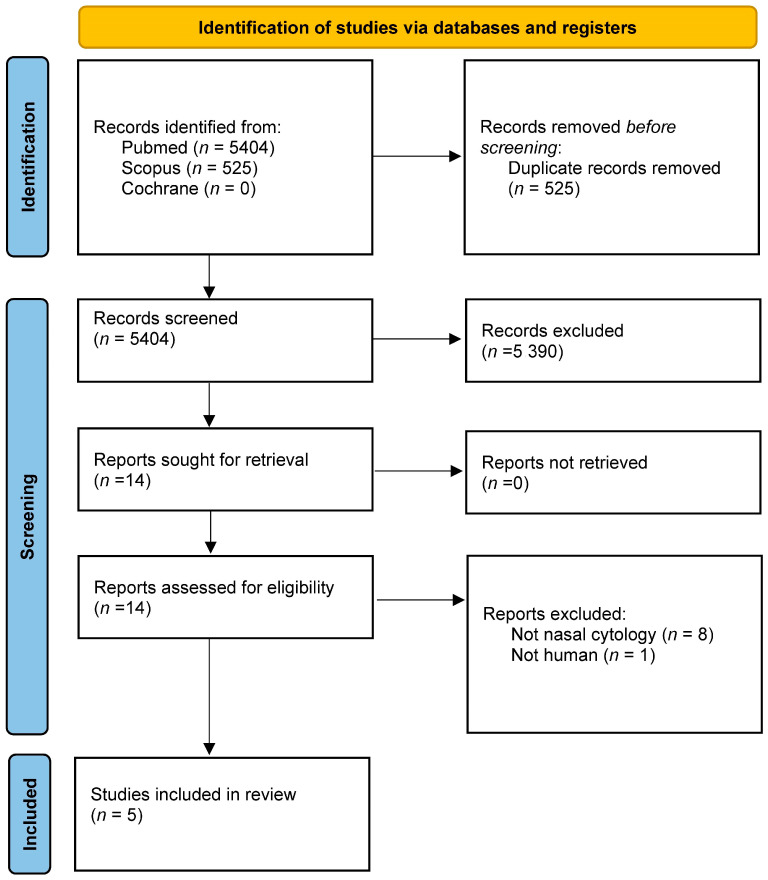
Review of the English literature performed through PubMed, Scopus, and Cochrane, accessed on 31 December 2022, according to Preferred Reporting Items for Systematic Reviews and Meta-Analyses (PRISMA) standards. Primary search was performed using the terms “nasal cytology”, “head neck”, and “cancer OR tumor OR radiotherapy”.

Five studies were included in qualitative synthesis: 3 assessed mucosal disorders after radiation therapy and 2 after total laryngectomy (Figure 1) [15,16,17,18,19]. Table 1 summarizes the studies about nasal cytology in HNC, while Table 2 reports the 9 excluded studies. No study evaluated nasal cytology as a diagnostic tool for sinonasal tumors.

## 3. Nasal Cytology in Head and Neck Cancer Chemoradiotherapy

RT represents one of the main therapeutic strategies for HNC treatment. In particular, RT can be chosen as a single-modality treatment for early-stage tumors as the first choice or as an alternative to surgery. On the other hand, the management of locally advanced tumors provides for a multimodal treatment that generally consists of surgery followed by adjuvant RT or CRT, or definitive CRT. Moreover, RT and CRT represent the main treatments for nasopharyngeal and oropharyngeal tumors [5]. According to modern radiation technology, multiple radiation beams are directed toward the tumor from different angles and planes, resulting in the delivery of the planned dose with only low fractions of radiation to the healthy surrounding tissues [29]. Over the past two decades, intensity-modulated radiation therapy (IMRT) has been increasingly used thanks to its ability to selectively deliver radiation beams to the primary tumor and the adjacent lymph node regions by decreasing the dose to the surrounding health structures. Therefore, it improved therapeutic results by reducing acute and/or chronic radiation toxicity effects [30].

Nevertheless, RT complications are well known and their incidence has decreased over the years but has not been eliminated yet. In particular, toxicity is more severe if RT is combined with chemotherapy (CT) because of a cytotoxic effect on rapidly growing cells, resulting in 77% incidence of acute adverse events of grade 3 or greater in patients who received CRT, compared to 34% in patients who received RT alone [31]. Common acute toxicity (defined as changes in the normal tissues occurring during RT or within 90 days from the end of RT) includes mucositis, perioral dermatitis, dysphagia, hoarseness, dysgeusia, and smell loss. Late toxicities (tissue changes occurring at least 90 days after RT end) are represented by osteoradionecrosis, xerostomia, fibrosis, thyroid dysregulation, sensorineural hearing loss, myelitis, and pharyngeal or laryngeal stenosis [32,33,34]. In the sinonasal district, due to the dysregulation of nasal mucociliary clearance (MCC), RT is responsible for a variety of disruptions and alterations [35].

One of the most common side effects of RT for HNC is radiation-induced chronic rhinosinusitis (CRSr), defined as a chronic disorder of sinonasal cavities caused by RT, which leads to the typical sinonasal symptoms that characterize common chronic rhinosinusitis (CRS): nasal obstruction, purulent nasal discharge, facial pain, hyposmia. These clinical features contribute to undermining the quality of life of patients affected by HNC and could hide the early signs of a possible recurrence of malignancy and lead to a delay in its identification [26]. The severity of CRSr has been attributed to the administered radiation dose: 40 Gy could cause an acute mucosal inflammatory reaction, whereas 60–70 Gy could be responsible for ischemic necrosis and shedding [26]. Furthermore, Cooper et al. demonstrated that late clinically relevant alterations rarely occurred with doses lower than 50 Gy, while oral and nasal ulcerations were extremely rare for doses < 65 Gy [36]. In a recent review, the percentage of patients affected by CRSr ranged from 7% to 86.1%, and the most common pathogens isolated in the CRSr group were Staphilococcus aureus, Streptococcus viridans, and Pseudomonas aeruginosa [35]. Moreover, Stoddard et al. found that Staphilococcus aureus and Pseudomonas aeruginosa represented the most common bacteria in sinonasal cultures of patients affected by CRSr and concluded that the bacteriology of CRSr seemed to be similar to the usual microorganism colonization of non-radiated patients [28].

Pathological mechanisms that lead to the alteration of nasal mucosa in radiated patients have been extensively clarified over the years. Lou et al. described an increased deposition of dense collagenous fibers into the lamina propria in the histology of patients who received RT for nasopharyngeal carcinoma (NPC). They also identified a gradual reduction in cytoplasmic volume in nasal epithelium cells in addition to localized areas of ciliary loss, intercellular and intracellular vacuolation, and metaplasia of epithelial cells to a stratified arrangement, leading to damaged MCC at the infundibular epithelium at a median time of 5.9 years after RT [23]. Pathologic examination conducted by Kuhar et al. on intraoperative samples of 15 patients affected by CRSr who underwent Functional Endoscopic Sinus Surgery (FESS) revealed an increased thickness of lamina propria, areas of ciliary loss, intercellular and/or intracellular vacuolization, and epithelial metaplasia [27]. Furthermore, they investigated the type and severity of epithelial modifications in patients affected by CRSr in comparison to those who suffered from chronic rhinosinusitis with nasal polyps (CRSwNP) or chronic rhinosinusitis without nasal polyps (CRSsNP). Compared to CRSsNP, CRSr showed a significant increase in epithelial squamous metaplasia, but not in eosinophil count or neutrophilic infiltrate. CRSsNP and CRSr also presented the same grade of overall mucosal inflammation, fibrosis, and basal membrane thickening. However, CRSr showed a decreased count of eosinophils and a lower basal membrane thickening than CRSwNP [27].

According to the timing of the onset of radiation toxicity effects, Gruber et al. showed that acute damage to the nasal epithelium included vacuolization of ciliated cells, increased cell secretions, and stromal edema. On the other hand, late radiation toxicity could be revealed by a combination of cellular necrosis, the presence of reactive oxygen species, and the infiltration of proinflammatory and profibrotic cytokines [37]. Such effects can be explained by pathological changes in non-epithelial cell populations (macrophages, fibroblasts, vascular endothelial cells). As a matter of fact, the inflammatory response underlying the epithelial disruption normally precedes morphological cell changes: this is carried out by submucosal and mucosal vasodilatation, local hypoxia, activation of macrophages, and endothelial cells. Therefore, intra- and intercellular signaling pathways regulate inflammatory mediator levels in resident tissue cells and inflammatory cells recruited from blood (e.g., NF-kB) [37].

A major contribution to the comprehension of nasal disorders after RT for HNC, leading to radiation-induced rhinitis, was given by the assessment of nasal epithelium changes through nasal cytology. Riva et al. performed cytological examination through nasal scraping in 10 radiated patients affected by HNC, excluding those who suffered from nasal cavity tumors, CRSwNP, and CRSsNP. The authors detected neutrophilic infiltrate in 70% of cases that persisted up to 1 month after the end of treatment, while mucous cell metaplasia was observed in 10% of patients during RT and disappeared 3 months after the end of therapy. On the other hand, they observed squamous cell metaplasia in 10% of patients only after the completion of RT, resulting in a possible role of radiation beams in determining long-term cytological changes in the nasal mucosa. Mean dose (Dmean) and near-maximum dose (D2%) to inferior turbinates were associated with neutrophilic rhinitis, while D2% to inferior turbinates was correlated to mucous cell metaplasia at the end of RT [18].

Late effects of RT consist of mucosal paleness, epithelial thinning, submucosal induration, and occasionally, mucosal ulceration and necrosis with exposure of underlying bone [36]. Other evidence of the long-term rule of RT for NPC in determining changes in nasal mucosa can be seen by means of cytology, demonstrating a neutrophil infiltrate in 40% of radiated patients affected by NPC compared to 13% of healthy subjects (control group). Squamous cell metaplasia and mucous cell metaplasia were found in 20% and 13% of radiated patients, respectively. No difference between patients and the control group was observed regarding eosinophils (found only in patients affected by allergic rhinitis), while no lymphocytes and mast cells were found in either group. Furthermore, no cytological atypia was seen in either healthy or radiated individuals. Finally, no statistically significant correlation between cytological changes and sinonasal symptoms was revealed [16].

The squamous and mucous cell metaplasia in radiated patients may have an effect on MCC, as the disruption of ciliated cells in addition to the increase in goblet cells (both effects of radiation toxicity on mucosal cells) could lead to a major mucus production with consequent endonasal stagnation and bacterial superinfection. All these findings highlighted that direct histopathological effects of RT were evident only during or shortly after irradiation, due to the extremely rapid capacity of mucosal cells to replicate themselves. Bussu et al. showed a significantly reduced MCC in radiated patients. In particular, they compared subjects who received external beam RT to those treated with brachytherapy for nasal vestibule squamous cell carcinoma. They found that patients treated with external beam RT showed a significantly impaired MCC, with a doubled main time for the transport of the stained marker compared to patients who underwent brachytherapy. Moreover, the authors observed neutrophil inflammation with mucous cell metaplasia in patients who underwent external beam RT and not in those who underwent brachytherapy [19].

A major role in restoring mucociliary function in patients who underwent RT for HNC could be played by FESS in order to re-establish the aeration and drainage of paranasal sinuses after radiation treatment. However, particular attention must be paid to performing endoscopic surgery in radiated patients because of the thinness and atrophy of the sinonasal mucosa (especially in the fontanelle area), leading to possible iatrogenic damage. Kamel et al. reported MCC deterioration for up to 6 months after RT, without subsequent restoration. They also performed nasal endoscopy that highlighted early RT effects such as hyperemia, and edema of the mucosa 2–6 weeks after RT, and late RT effects represented by crusting, scarring, adhesions, atrophy of turbinates, ostial widening, and choanal stenosis [24]. In these cases, FESS plays a substantial role in restoring sinonasal aeration, especially in cases of post-radiation choanal stenosis and/or atresia.

To summarize, RT for HNC may be responsible for radiation-induced rhinitis and/or chronic rhinosinusitis that may persist after the end of treatment, affecting the patient’s quality of life. In particular, RT may induce neutrophilic inflammation and squamous or mucous cell metaplasia. However, a standardized line of therapy for these disorders is still missing, and the execution of nasal cytology for all patients who undergo RT should help physicians understand the underlying pathogenetic mechanisms and choose the best treatment for each patient.

## 4. Nasal Cytology in Head and Neck Cancer Surgery

HNC surgery may lead to alterations of sinonasal anatomy or changes in nasal airflow, leading to potential disorders of nasal mucosa. Cytological examination has never been conducted after surgery for sinonasal cancer, but some studies have been performed in laryngectomees [17,38]. Total laryngectomy results in a complete and permanent separation of the upper and lower respiratory tract, excluding the nose from respiration and regular airflow. This prevents the physiological heating, moistening, and filtering of the inspired air, leading to unfiltered and non-conditioned air flowing directly into the trachea, associated with smell and taste loss [39].

Although some studies reported higher rates of respiratory tracts infections in laryngectomized patients, most of them agreed on significantly higher microbial colonization of both nasal cavities and trachea in laryngectomees with little or no clinical and cytological signs of inflammation [15,38,40,41]. Skoloudik et al. reported cytological evidence of rare and low-grade inflammation of the nasal mucosa and no clinical evidence of suppurative rhinosinusitis in the first 3 years after total laryngectomy [15].

One study showed that 92% of laryngectomized patients had Gram-positive polimicrobic flora in their nasal cavities, while 48% of them had it in the trachea. Some non-fermenters Gram-negative bacteria were also found [38]. Skoloudik et al. mostly found Staphylococcus epidermidis, Staphylococcus aureus, Pseudomonas aeruginosa, Proteus vulgaris, Proteus mirabilis, and Haemophilus influenzae in nasal cavities [15], while Cvetnic et al. reported Staphylococcus epidermidis, Proteus mirabilis, and Pseudomonas aeruginosa as main findings at 8 and 30 days after surgery [40]. The latter also reported that sterile nasal cavities were observed in 25% of cases 8 days after total laryngectomy and in 56% after 30 days. The reason is probably related to the broad-spectrum antibiotics administered in the immediate post-operative time [40]. Kramp et al. most frequently found Staphylococcus aureus and Candida albicans, and evaluated the effects of tracheostomal protective devices, finding evidence of reduced colonization in those patients who wore such devices [41]. 

An important barrier to nasal infections and colonization is mucociliary transport. Some authors reported an increased function in the early years after total laryngectomy, and it could be the reason for the less frequent inflammatory changes after total laryngectomy [15,20,42]. In particular, Maurizi et al. showed an increased MCC as early as 60 days after the surgery, probably due to an increase in endonasal temperature and humidity, and the reduction in the watery component. Nasal MCC decreased 5 years after surgery, probably because of the saprophytic bacteria colonization. Moreover, an increase in tracheobronchial MCC velocity due to an early hypersecretory phase was observed, protecting tracheal mucosa from unfiltered and unconditioned air [43]. 

Deniz et al. compared MCC in patients who had undergone total laryngectomy less than 2 years before to patients operated on more than 2 years before and a control group of healthy individuals. They found significantly faster MCC in patients operated on less than 2 years before compared to the control group, while MCC was significantly slower in patients operated on more than 2 years before compared to the control group [42]. These results are consistent with those of Maurizi et al., finding an immediate and significant increase in clearance right after surgery, followed by a slower clearance some years after surgery. 

Moore-Gillon et al. performed a study including 10 patients about to undergo a total laryngectomy and 23 patients who had undergone the procedure 1 to 15 years before. The study showed how mucociliary transport, measured through saccharin clearance, was significantly faster in laryngectomized patients [20]. Furthermore, Todisco et al. observed an MCC increase 2 months after total laryngectomy, while this result was not found in any of the patients who had undergone a partial laryngectomy [21].

Cytological alterations in nasal and tracheal mucosa have been investigated in a few studies, which suggested that the separation of the two tracts leads to significant cytological changes in both districts [15,17,25]. The healthy nasal epithelium is composed of squamous cells in the nasal vestibule. Moving from anterior to posterior, the nasal epithelium changes progressively to a transitional epithelium and then to a pseudostratified columnar epithelium [44].

Experiments performed on living rabbits showed a change in the nasal epithelium after unilateral naris closing. In particular, the results showed an increased number of goblet cells and the changing of the transitional epithelium into a ciliated epithelium on the closed side at histology, whereas the open side, subjected to a faster and larger airflow, underwent a significant reduction in the number of goblet cells and an extension of the squamous epithelium area. Paranasal sinuses mucosa did not change despite the surgical closing of the naris, suggesting that the airflow exposure has an impact on these histological changes [44]. The human respiratory nasal epithelium after a total laryngectomy is believed to undergo similar changes to those happening to the surgically closed naris. 

Skoloudik et al. compared a group of 30 patients with total laryngectomy to a control group of 30 healthy individuals. The cytological examination showed significantly higher hyperplasia of the basal zone cells in the nasal cavities of laryngectomees (73% of laryngectomees vs. 23% of healthy individuals), while the results for squamous cell metaplasia did not reach statistical significance. Inflammatory changes (neutrophilic granulocytes) in the nasal samples were not found in any of the laryngectomees, while they were found in 57% of healthy individuals. No statistically significant difference between patients undergoing RT and patients who did not receive it was observed, since the nose was not included in the radiation volumes for such tumors [15]. 

In the study by Moore-Gillon et al., besides the saccharin test for MCC, the patients also underwent a nasal mucosa biopsy. The results showed a transitional epithelium prior to surgery, while the main finding after surgery was a densely ciliated mucosa with an abundance of mucus [20].

Riva et al. analyzed the cytological changes in nasal and tracheal mucosa in 25 laryngectomees. The authors found nasal mucous cell metaplasia in 20% of patients, versus 0% in the healthy control group. Squamous cell metaplasia was absent in the two groups enrolled in the study. No significant difference was found in terms of neutrophilic and eosinophilic infiltration. This study also analyzed tracheal cytology in the same group of laryngectomized patients. Tracheal squamous cell metaplasia was found in 24% of patients, and 32% of them had neutrophilic infiltrate. No control group was used for this analysis, since minimally invasive tracheal scraping was possible only in laryngectomized patients. Only four (16%) laryngectomees showed normal nasal mucosa and only two (8%) of them showed normal tracheal mucosa [17]. It is not possible to compare the tracheal data with others in the literature, being the only study with a focus on tracheal cytology found in the literature.

To summarize, total laryngectomy may induce mucous cell metaplasia, but not inflammatory changes of nasal mucosa. Moreover, bacteria can be observed in some cases without infection signs. Such alterations are likely due to the absence of nasal airflow and changes in MCC.

## 5. Discussion and Future Perspectives

Both surgical and medical treatments for HNC can determine profound changes in the physiology of the upper and lower airways (Table 3).

Nasal cytology, which is abundantly used in the research field of rhinology, has not been investigated as thoroughly in the oncological field, given the scarcity of studies found in the literature. Nasal cytology could be adopted as a standardized pre-treatment assessment in HNC patients, followed by post-treatment cytological evaluation in order to better understand the inflammatory and epithelial changes taking place in the nose. This can have implications for the stratification of the risk of infections and colonization of the airways. In particular, nasal cytology can identify mucosal disorders in laryngectomees and after radiation therapy. The main limitation of this review is the low number and heterogeneity of studies present in the literature and included in this qualitative analysis.

Further studies with larger samples are necessary to better understand cytological changes after HNC treatments and thus help physicians adequately treat nasal complaints of HNC patients in daily clinical practice. Nasal irrigation, topical antibiotics, corticosteroids, and hyaluronic acid are the main available therapies for rhinitis. Nasal cytology may help the physician to choose the treatment that best suits each patient. In particular, topical corticosteroids can be administered in patients with inflammatory infiltration and associated with antibiotics if infectious bacterial rhinitis is present. Hyaluronic acid can represent the treatment for nasal complaints due to epithelial changes and may be administered in association with other treatments. Finally, nasal irrigations are used in every kind of rhinitis.

Future studies evaluating radiation-induced rhinitis will allow us to identify subjects prone to developing acute and late nasal toxicities and to intervene promptly. Moreover, nasal cytology may help to find the best strategy to solve nasal complaints after total laryngectomy.

## Figures and Tables

**Table 1:** Nasal cytology in head and neck cancer: literature review.

Author, Year, Country	Study Design	Number of Patients	Sex	Age, Mean and Range (Years)	Tumor (Site and Stage)	Treatments	Measurements	Time of Assessment	Results
Skoloudik et al., 2009, Czech Republic [15]	Prospective	Study group: *n* = 30 Control group: *n* = 30	Study group M: 24 (80%) F: 4 (20%) Control group M: 25 (83%) F: 5 (17%)	Study group: 64 (47–78) Control group: 47 (20–74)	Larynx (stage NR)	Total laryngectomy: - Without adjuvant RT (*n* = 7) - With adjuvant RT (*n* = 22) - With adjuvant CT-RT (*n* = 1)	- Nasal endoscopy - Nasal cytology - Nasal microbiological examination	NR	- Higher hyperplasia of the basal zone cells in laryngectomees (73% in the study group, 23% in the control group) - No significant difference concerning squamous cell metaplasia (17% in the study group, 20% in the control group) - Higher percentages of mucosal inflammatory changes in the control group - 27% of laryngectomees presented bacteria in the nasal mucosa without inflammatory changes - No correlation between cytological changes and adjuvant RT
Riva et al., 2015, Italy [16]	Cross-sectional	Study group: *n* = 30 Control group: *n* = 30	Study group M: 24 (80%) F: 6 (20%) Control group M: 20 (67%) F: 10 (33%)	Study group: 53.53 (37–75) Control group: 52.35 (42–76)	Nasopharynx (stage I–IV)	2D-RT (*n* = 5), 3D-CRT (*n* = 5), IMRT (*n* = 20) RT dose: 69.34 ± 1.17 Gy - Concurrent (CT-RT) + adjuvant CT (*n* = 4) - Induction CT + concurrent CT-RT (*n* = 22) *Dose to nasal cavities*: NR	- Subjective nasal symptoms - Nasal endoscopy - Nasal cytology	59 (21–124) months after RT	- Higher percentage of rhinorrhea, nasal obstruction, mucosal hyperemia, and presence of nasopharyngeal secretions in the study group - Higher percentage of neutrophilic inflammation and squamous or mucous cell metaplasia in the study group - No cytological atypia - No correlation between cytological changes and symptoms, endoscopic findings, age, smoking, tumor stage - No significant difference between different radiation techniques and radiation dose
Riva et al., 2017, Italy [17]	Cross-sectional	Study group: *n* = 25 Control group: *n* = 25	Study group M: 22 (88%) F: 3 (12%) Control group M: 19 (76%) F 6 (24%)	Study group: 68.76 (50–83) Control group: 62.64 (48–76)	Larynx (stage II–IV)	Total laryngectomy: - Without adjuvant RT (*n* = 15) - With adjuvant CT-RT (*n* = 2) *Dose to nasal cavities*: NR	- Subjective nasal symptoms - Nasal endoscopy - Nasal cytology - Biopsy of inferior turbinate (light microscope views)	52 (26–97) months after treatment	- Mucous cell metaplasia in 20% of laryngectomees - Submucosal stromal fibrosis in all patients and submucosal inflammatory infiltrate in 1 case (9%) at histological examination - No correlation between cytological changes and symptoms, endoscopic findings (turbinate hypertrophy, mucosal hyperemia, nasal secretions), age, smoking, tumor stage, adjuvant RT
Riva et al., 2019, Italy [18]	Prospective	*n* = 10	M: 10 (100%)	56.90 (39–72)	Nasopharynx (*n* = 3), oral cavity (*n* = 3), parotid gland (*n* = 3), primary unknown (*n* = 1) (stage I–IV)	Surgery (*n* = 8) Concurrent CT-RT (54–70 Gy) (*n* = 5) Induction CT + concurrent CT-RT (*n* = 1) *Dose to nasal cavities*: - Mean dose (Dmean) to nasal cavities 13.59 ± 17.74 Gy - Near-maximum dose (D2%) to nasal cavities 26.79 ± 31.80 Gy - Mean dose (Dmean) to inferior turbinate 18.90 ± 24.08 Gy - Near-maximum dose (D2%) to inferior turbinate 26.46 ± 31.43 Gy	- Nasal endoscopy - Nasal cytology - NOSE scale and subjective nasal symptoms - Mean dose (Dmean) and near-maximum dose (D2%) to nasal cavities and inferior turbinates	Before (T0), at mid-course (T1), and at the end (T2) of RT, 1 and 3 months after RT (T3 and T4)	- Nasal symptoms and endoscopic findings peaked at the end of RT (T2) (rhinorrhea in 70% of cases, crusting in 40%) - Nasal cytology showed that radiation-induced rhinitis with neutrophils and sometimes bacteria occurred in 70% of cases and persisted after 1 month. Mucous cell metaplasia appeared in 10% of patients during RT and disappeared after 3 months. Squamous cell metaplasia was observed in 10% of cases only after the end of RT - No significant increase in NOSE total score at T2 - Significant correlation between Dmean and D2% to inferior turbinates and neutrophilic rhinitis at T2, between D2% to inferior turbinates and mucous cell metaplasia at T2
Bussu et al., 2020, Italy [19]	Cross-sectional	Study group 1 (IRT): *n* = 10 Study group 2 (EBRT): *n* = 8 Control group: *n* = 10	Study group 1 M: 6 (60%) F: 4 (40%) Study group 2 M: 6 (75%) F: 2 (25%) Control group M: 7 (70%) F: 3 (30%)	Study group 1: 56 (53–79) Study group 2: 66 (58–79) Control group: 58 (50–76)	Nasal vestibule (stage I–IV)	IRT (*n* = 10), EBRT (*n* = 8) IRT dose: 44 Gy total dose (3 Gy per fraction, except first and last fraction 4 Gy) EBRT dose: 40 Gy *Dose to nasal cavities*: NR	- Rhinomanometry and nasal decongestion test - Olfactometry - Nasal cytology - Nasal endoscopy - MCC (saccharin test) - NOSE scale and subjective nasal symptoms	Study group 1: 34 (24–70) months after interstitial IRT Study group 2: 64 (40–83) months after intensity-modulated EBRT	- Significant difference in NOSE scale score (higher in patients of study group 2) - Higher nasal flow and lower resistance at rhinomanometry in study group 2 - No significant difference between study group 1 and control group concerning nasal flow and nasal resistance - Reduced MCC in study group 2 - Better scores of TDI in study group 1 than in study group 2 - Significant difference in distribution of cytological patterns between the two study groups - Mucous cell metaplasia associated with neutrophilic nasal flogosis in 40% of patients of study group 2 - Only 1 case of eosinophilic nasal flogosis in study group 1 and 1 case in the control group - No eosinophilic nasal flogosis in study group 2

Abbreviations: 2D-RT, two-dimensional radiotherapy; 3D-CRT, three-dimensional conformal radiotherapy; CT, chemotherapy; CT-RT, chemora-diotherapy; EBRT, external beam radiotherapy; F, female; Gy, gray; IMRT, intensity-modulated radiation therapy; IRT, interventional radiotherapy; M, male; MCC, mucociliary clearance; NOSE, Nasal Obstruction Symptom Evaluation; NR, not reported; RT, radiotherapy; TDI, threshold discrim-ination identification.

**Table 2:** Excluded studies after assessment for eligibility.

Author, Year	Citation
Moore-Gillon, V., 1985	[20]
Todisco et al., 1986	[21]
Panossian et al., 1992	[22]
Chen et al., 1999	[23]
Kamel et al., 2004	[24]
Karaca et al., 2010	[25]
Su et al., 2014	[26]
Kuhar et al., 2017	[27]
Stoddard et al., 2019	[28]

**Table 3:** Nasal cytological changes after head and neck surgery or radiotherapy.

Treatment	Early Effects	Late Effects
Surgery (total laryngectomy)	- Increase in nasal mucociliary clearance (up to 5 years since surgery)	- Decrease in nasal mucociliary clearance (5 years after surgery) - Hyperplasia of the basal zone cells - Increased cell secretion
Radiation therapy	- Vacuolization of ciliated cells - Increased cell secretion - Stromal edema - Neutrophilic infiltrate - Mucous cell metaplasia	- Cellular necrosis - Infiltration of proinflammatory and profibrotic cytokines - Increased thickness of lamina propria - Intercellular and/or intracellular vacuolization - Squamous or mucous cell metaplasia - Basal membrane thickening - Decrease and deterioration of nasal mucociliary clearance

## Data Availability

The review was not registered and a protocol was not prepared.

## Supplementary Materials

The following supporting information can be downloaded at: https://www.mdpi.com/article/10.3390/diagnostics13152480/s1. Reference [45] is cited in Supplementary Materials.
